# D. W. Young, M.D., Aurora, Ill.

**Published:** 1874-09-15

**Authors:** 


### OBITUARY.

D. W. Young, M. D., of Aurora, Ill., died on the evening of Sept. 8th, 1874. He had suffered more or less from disease of the digestive organs for several years, but was not entirely disabled from business until a few weeks before his death. Dr. Young was a practitioner of ability and industry, and a very valuable and enterprising citizen. He was an active supporter of the social organizations of the profession, having recently occupied the position of President of the State Medical Society. He always took an active part in the municipal affairs of his city, and served faithfully in the office of Mayor a number of years.

